# Palliative Gastrectomy vs. Gastrojejunostomy for Advanced Gastric Cancer: A Systematic Review and Meta-Analysis

**DOI:** 10.3389/fsurg.2021.723065

**Published:** 2021-11-26

**Authors:** Chunfang Lin, Haibo Fan, Wenjun Chen, Lingzhi Cui

**Affiliations:** ^1^Department of General Surgery, The Second Hospital of Shanxi Medical University, Taiyuan, China; ^2^Department of Targeted Therapy, Shanxi Cancer Hospital, Taiyuan, China

**Keywords:** gastric cancer, gastrojejunal bypass, palliative resection, complications, morbidity, mortality

## Abstract

**Background:** Advanced gastric cancer is the fifth leading cause of cancer-related deaths. Patients with metastatic advanced gastric cancer commonly develop a gastric outlet obstruction that considerably worsens their quality of life. Surgical interventions such as gastrojejunostomy and palliative gastrectomy are commonly administered to alleviate this obstruction. However, whether one intervention is better than another at improving morbidity- and mortality-related outcomes is unclear. Thus, in this meta-analysis, we compare outcomes of palliative gastrectomy and gastrojejunostomy (overall hospital stay length, time to oral intake, survival, and complication rates) in patients with metastatic advanced gastric cancer to identify the best procedure.

**Objective:** To compare morbidity and mortality outcomes of palliative gastrectomy and gastrojejunostomy in patients with metastatic advanced gastric cancer.

**Methods:** We followed the PRISMA guidelines to systematically search Web of Science, EMBASE, CENTRAL, Scopus, and MEDLINE for relevant studies. We conducted a random-effects meta-analysis to find differential outcomes between palliative gastrectomy and gastrojejunostomy among variables such as time to oral intake, overall hospital stay length, complication rates, and survival in patients with metastatic advanced gastric cancer.

**Results:** From 963 studies, we found 7 eligible studies with 642 patients (70.3 ± 4.7 years) who had undergone palliative gastrectomy or gastrojejunostomy. Our meta-analysis revealed an insignificant (*p* > 0.05) differences in terms of overall survival duration (Hedge's *g*, 1.22), complication risks (odds ratio, 1.35), and time to oral intake (*g*, 0.62) and hospital stay length (*g*, 0.12) between patients undergoing gastrojejunostomy and palliative gastrectomy.

**Conclusion:** In this present study we observed no statistically significant differences in terms of morbidity and mortality outcomes after palliative gastrectomy and gastrojejunostomy in patients with metastatic advanced gastric cancer. Therefore, no conclusions can be drawn for the variables evaluated. This study provides a preliminary overview of the risks associated with gastrojejunostomy and palliative gastrectomy to help gastroenterologists manage patients with metastatic advanced-stage gastric cancer.

## Introduction

Gastric cancer is the fifth most common cancer worldwide (1). According to the American Cancer Society, the severity of gastric cancer depends on the extent of the cancerous tissue's growth across the gastrointestinal layers and/or into the adjacent digestive organs (2). Epidemiological studies have widely reported a high incidence of gastric cancer worldwide (15.5 per 100,000 people) (3), and the Global Burden of Disease study estimates that almost 785,000 patients die annually due to gastric cancer (4, 5).

Most patients with gastric cancer are diagnosed during advanced stages of the disease (6) with extremely poor morbidity- and mortality-related outcomes (7, 8). Patients with metastatic advanced-stage gastric cancer also present gastrointestinal complications, with gastric outlet obstruction being the most common one (9, 10). According to Khullar and DiSario (11), patients with gastric outlet obstruction can have symptomatic manifestations (vomiting, malnutrition, nausea, and dehydration) that depend upon the site (pyloric or proximal duodenum) and the extent of the obstruction. These symptoms impair the patients' ability to receive appropriate oral palliative treatment and considerably worsen their quality of life (12). Surgical interventions such as gastrojejunostomy and palliative gastrectomy have been widely recommended to alleviate symptoms and improve the patients' quality of life (9, 13, 14). Gastrojejunostomy involves the creation of an anastomosis (a bypass) between the stomach and the jejunum to bypass the gastric outlet obstruction (15), and palliative gastrectomy involves the resection of the gastric outlet obstruction to improve stomach emptying (14).

Retrospective cohort studies have compared morbidity- and mortality-related outcomes in patients with advanced gastric cancer undergoing palliative gastrectomy or gastrojejunostomy (9, 13, 14, 16–19). However, the evidence for the impact of these surgical interventions on the survival and complication rates of the patients with advanced gastric cancer is contradictory. Some studies have shown an increased survival in patients undergoing palliative gastrectomy compared with those undergoing gastrojejunostomy (9, 16, 18). However, Okumura et al. (17) reported longer survival times for patients receiving gastrojejunostomy. Similarly, some studies have reported that palliative gastrectomy was associated with higher complication risks (9, 14, 18), while others have reported higher risks for gastrojejunostomy (13, 16, 17). To the best of our knowledge, no systematic review or meta-analysis has compared the morbidity- and mortality-related impact of palliative gastrectomy and gastrojejunostomy in patients with metastatic advanced gastric cancer.

Thus, in this systematic review and meta-analysis, we compare overall survival, hospital stay length, complication rates, and times to oral intake in patients undergoing either palliative gastrectomy or gastrojejunostomy. Our findings should be valuable to gastroenterologists managing patients with metastatic advanced gastric cancer.

## Methods

We adhered to PRISMA (Preferred Reporting Items for Systematic Reviews and Meta-Analyses) guidelines (20) while performing this meta-analysis.

### Data Search Strategy

We searched for publications in Web of Science, MEDLINE, CENTRAL, EMBASE, and Scopus (from inception until February 2021) using MeSH keywords including “Gastric cancer,” “gastrectomy,” “palliative gastrectomy,” “gastrojejunostomy,” “morbidity,” and “mortality.” We also searched the bibliography section of the included studies manually to identify further relevant studies. Our inclusion criteria were the following,

a) Studies comparing overall hospital stay length, time to oral intake, and overall survival in patients with advanced gastric cancer undergoing palliative gastrectomy or gastrojejunostomy.
b) Studies comparing complication rates in patients with advanced gastric cancer undergoing palliative gastrectomy or gastrojejunostomy.
c) Studies with human participants.
d) Case-control studies, prospective cohort trials, or retrospective cohort trials.
e) Studies published in peer-reviewed scientific journals.
f) Studies published in English.

The screening of the studies was independently performed by two reviewers. Disagreements were resolved by discussion with a third independent reviewer.

### Quality Assessment

We used the Newcastle Ottawa scale (21) to appraise the risk of bias in the included studies. This tool evaluates bias due to selective reporting, confounding factors, measurement of outcomes, and incomplete data availability. Two reviewers independently assessed the methodological quality of the studies, and a third reviewer intervened to arbitrate in case of disagreements.

### Data Analysis

A within-group meta-analysis was performed using the Comprehensive Meta-analysis version 2.0 (22). We conducted the meta-analysis based on the random-effects model (23). Odds ratios were calculated to evaluate the complication risks in patients undergoing either palliative gastrectomy or gastrojejunostomy. We also calculated weighted effect sizes (Hedge's *g*) to evaluate the outcomes (overall hospital stay length, time to oral intake, and survival). We computed *I*_2_ statistics to assess the heterogeneity among studies. We considered values between 0 and 25% as having negligible heterogeneity, those between 25 and 75% as having moderate heterogeneity, and those ≥75% as having substantial heterogeneity (24). We used the method listed by Hozo et al. (25) to convert medians and ranges (i.e., mentioned in the descriptive statistics of the included studies) into means and standard deviations for data analysis, respectively. In addition, we used Duval and Tweedy's trim and fill procedure to evaluate publication bias (26). The significance level for this study was determined at 5%.

## Results

**Figure 1:**
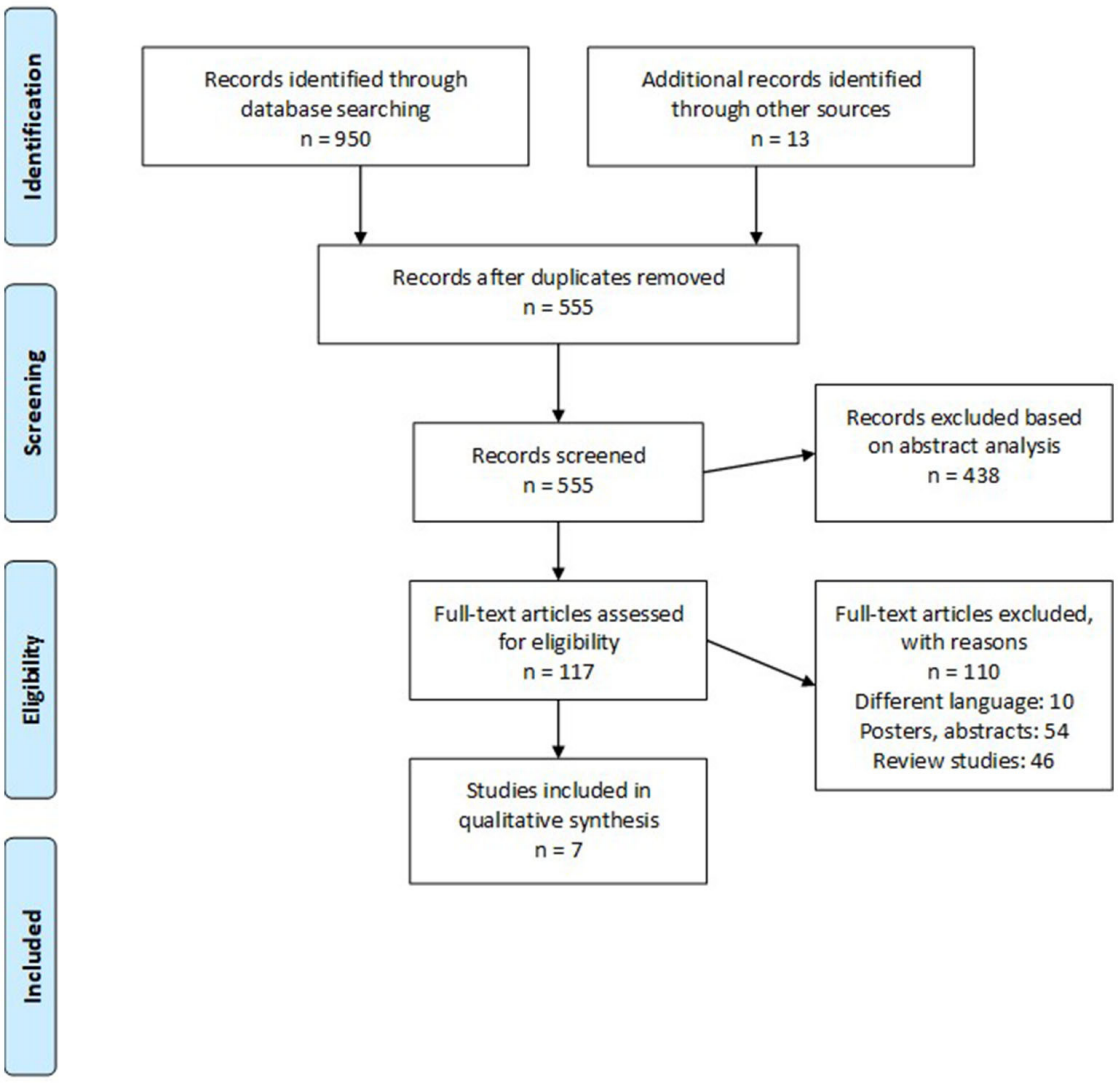
PRISMA flowchart.

We retrieved 950 studies after our initial database search. We identified an additional 13 studies by screening the reference sections of the included studies. Only seven retrospective cohort studies (9, 13, 14, 16–19) fit our inclusion criteria (Figure 1). Table 1 summarizes the extracted data.

**Table 1:** Details of the included studies.

**Study**	**Country**	**Type of study**	**Sample descriptive**	**Age (M ± S.D years)**	**Location**	**Type of surgery**	**Patients with gastric outlet obstruction (score)**	**Patients with peritoneal seeding**	**Patients with distant metastases**	**Time to oral intake (days)**	**Postoperative hospital stay (days)**	**Complications (%)**	**Survival (days)**
Chen et al. (16)	China	Retrospective cohort study	GJ: 89 (22W, 67M)	–	Distal	–	GJ (0: 30, 1: 59)	GJ: 77	–	GJ: 5.8 ± 1.5	GJ: 10.8 ± 3.6	GJ: 19.2%	GJ: 212.9 ± 30.4
			PG: 110 (37W, 73M)				PG (0: 13, 1: 39)	PG: 85		PG: 6.1 ± 2.1	PG: 14.3 ± 8.2	PG: 17.3%	PG: 456.2 ± 60.8
Matsubara et al. (13)	Japan	Retrospective cohort study	GJ: 29 (11W, 18M)	GJ: 70.3 ± 7.6	Distal, proximal	Distal/ proximal/total gastrectomy	–	–	–	–	GJ: 22.4 ± 21.9	GJ: 12%	-
			PG: 81 (27W, 54M)	PG: 66.4 ± 11.4							PG: 26.5 ± 17.4	PG: 22%	
Omori et al. (14)	Japan	Retrospective cohort study	GJ: 19 (6W, 13M)	GJ: 78	Distal	Billroth I, Billroth II or Roux–en–Y reconstruction	GJ (0: 5, 1: 13, 2: 15, 3: 17)	GJ: 14	GJ: 2	GJ: 67	–	GJ: 0 %	GJ: 86
			PG: 40 (7W, 33M)	PG: 77			PG (0: 7, 1: 7, 2: 1, 3: 4)	PG: 26	PG: 12	PG: 140		PG: 25%	PG: 145
Sahakyan et al. (19)	Armenia	Retrospective cohort study	GJ: 42 (11W, 31M)	–	–	Subtotal/total gastrectomy	–	–	–	–	–	GJ: 21.5%	GJ: 120
			PG: 70 (32W, 38M)									PG:18.6%	PG: 210
Okumura et al. (17)	Japan	Retrospective cohort study	GJ: 25 (8W, 17M)	GJ: 70	Distal	–	GJ (0: 13, 1: 10, 2: 2, 3: 0)	GJ: 19	GJ: 6	–	–	GJ: 32%	GJ: 264
			PG: 18 (8W, 10M)	PG: 74			PG (0: 11, 1: 3, 2: 1, 3: 3)	PG: 13	PG: 4			PG: 11.1%	PG: 249
Keränen et al. (9)	Finland	Retrospective cohort study	GJ: 21 (11W, 10M)	GJ: 69	–	–	GJ (0: 10, 1: 2, 2: 6, 3: 3)	–	–	GJ: 5.2 ± 2.0	GJ: 11.7 ± 6.6	GJ: 10%	GJ: 237.2 ± 186.2
			PG: 26 (9W, 17M)	PG: 70			PG (0: 9, 1: 10, 2: 6, 3: 1)			PG: 5.5 ± 2.3	PG: 9 ± 3.4	PG: 35%	PG: 822.7 ± 796.4
Ouchi et al. (18)	Japan	Retrospective cohort study	GJ: 15 (1W, 14M)	GJ: 64.6	Distal	Distal/total gastrectomy	–			–	–	GJ: 20%	GJ: 120
			PG: 64 (20W, 44M)	PG: 64.3								PG: 34%	PG: 300

*M, mean; SD, standard deviation; W, women; M, men; PG, palliative gastrectomy; GJ, gastrojejunostomy*.

### Participant Information

Data from 649 patients (210W, 439M) were included in the 7 studies. We found 240 patients (70W, 170M) who underwent gastrojejunostomy and 409 (140W, 269M) who underwent palliative gastrectomy.

The average age of the participants was 70.3 ± 4.7 years. The average age of patients undergoing gastrojejunostomy was 70.3 ± 4.8 years and the average age of those undergoing palliative gastrectomy was 70.3 ± 5.2 years. Two studies failed to report the age distribution of their sample (16, 19).

### Quality Assessment for Cohort Studies

Supplementary Table 1 shows the Newcastle Ottawa scale results for the risks of bias. The overall risk was low and is demonstrated in Supplementary Figure 1.

### Publication Bias

**Figure 2:**
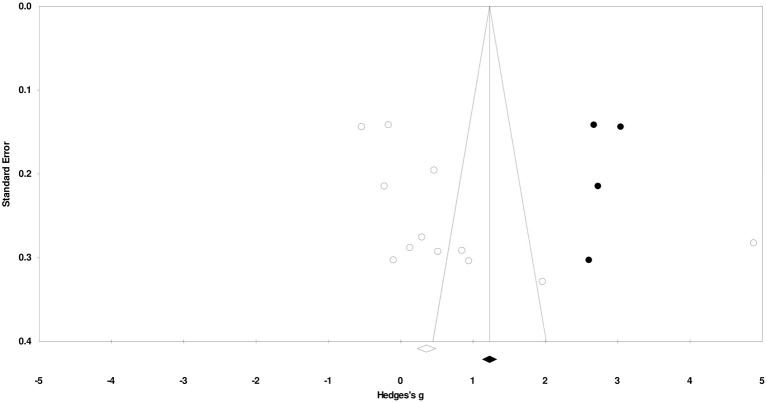
Publication bias by Duval and Tweedy's trim and fill method.

Duval and Tweedy's trim and fill method identified four missing studies on the right side of the mean effect. The overall random effect models determined the point estimates and the 95% confidence intervals for all the combined studies at 0.74 (0.01–1.48). After using the trim and fill method, the imputed point estimates were estimated at 1.25 (0.46–2.05). Figure 2 reports the publication bias results.

### Meta-Analysis Report

#### Time to Oral Intake

**Figure 3:**
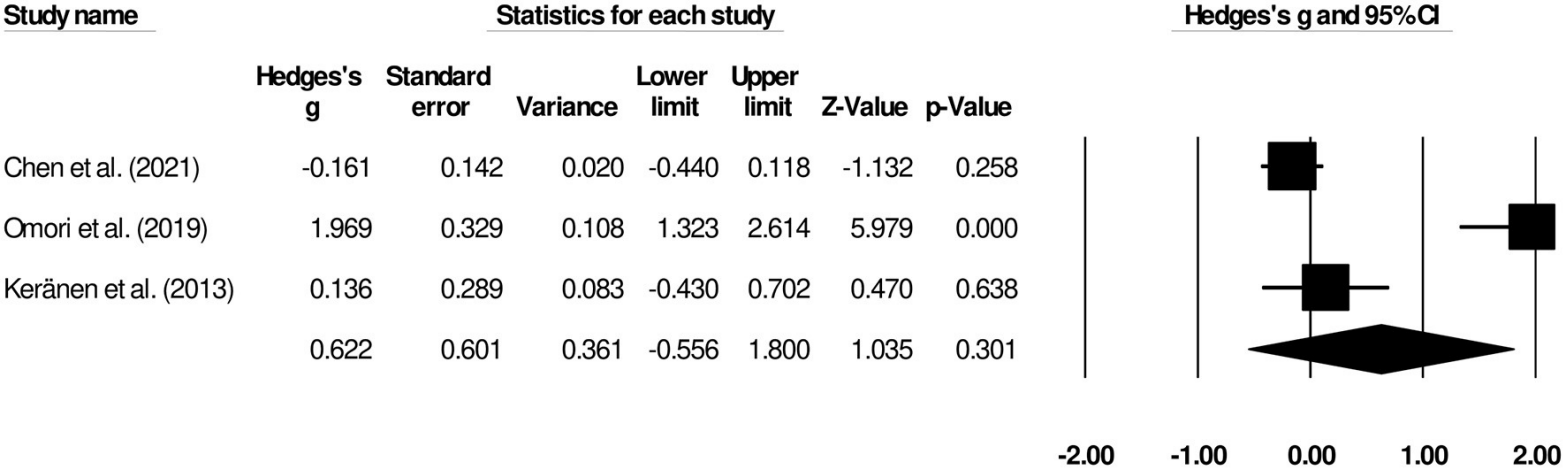
Forest plot for studies comparing the overall time to oral intake for patients undergoing gastrojejunostomy or palliative gastrectomy.

The time to oral intake was reported by three studies (9, 14, 16). We observed a *moderate* insignificant effect on time to oral intake between patients undergoing palliative gastrectomy and gastrojejunostomy (Figure 3) (Hedge's *g*, 0.62; 95% CI, −0.55 to 1.80; *p* = 0.30) with negligible heterogeneity (*I*^2^, 17.5%).

#### Duration of Hospital Stay

**Figure 4:**
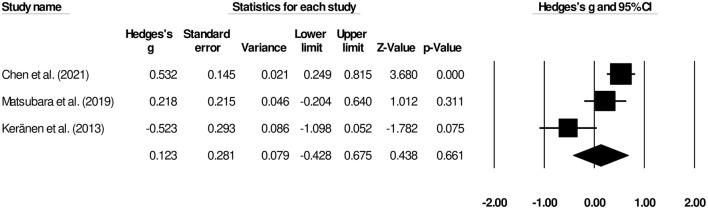
Forest plot for studies evaluating the overall hospital stay length for patients undergoing gastrojejunostomy or palliative gastrectomy.

Three studies reported the overall hospital stay length (9, 13, 16). We observed a *small* insignificant effect on the duration hospital stay between patients undergoing palliative gastrectomy and gastrojejunostomy (Figure 4) (Hedge's g, 0.12; 95% CI, −0.42 to 0.67; *p* = 0.66) with negligible heterogeneity (*I*^2^, 41.8%).

#### Complications

**Figure 5:**
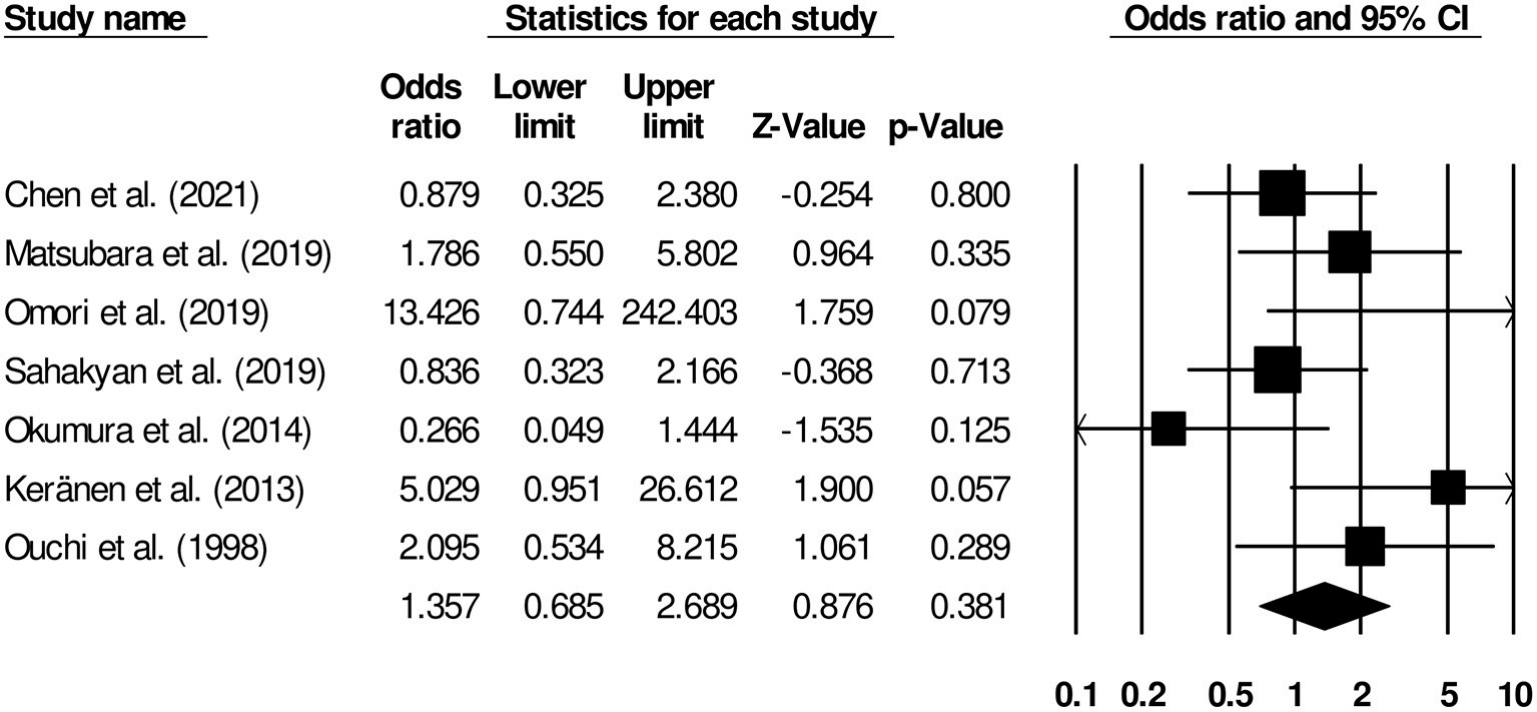
Forest plot for studies comparing complication rates in patients undergoing gastrojejunostomy or palliative gastrectomy.

The complication rates of patients undergoing gastrojejunostomy or palliative gastrectomy were reported by seven studies (9, 13, 14, 16–19). We observed insignificant differences in terms of risk of complication between patients undergoing palliative gastrectomy and gastrojejunostomy (Figure 5) (odds ratio, 1.35; 95% CI, 0.68–2.68; *p* = 0.38) with negligible heterogeneity (*I*^2^, 16.2%).

#### Survival

**Figure 6:**
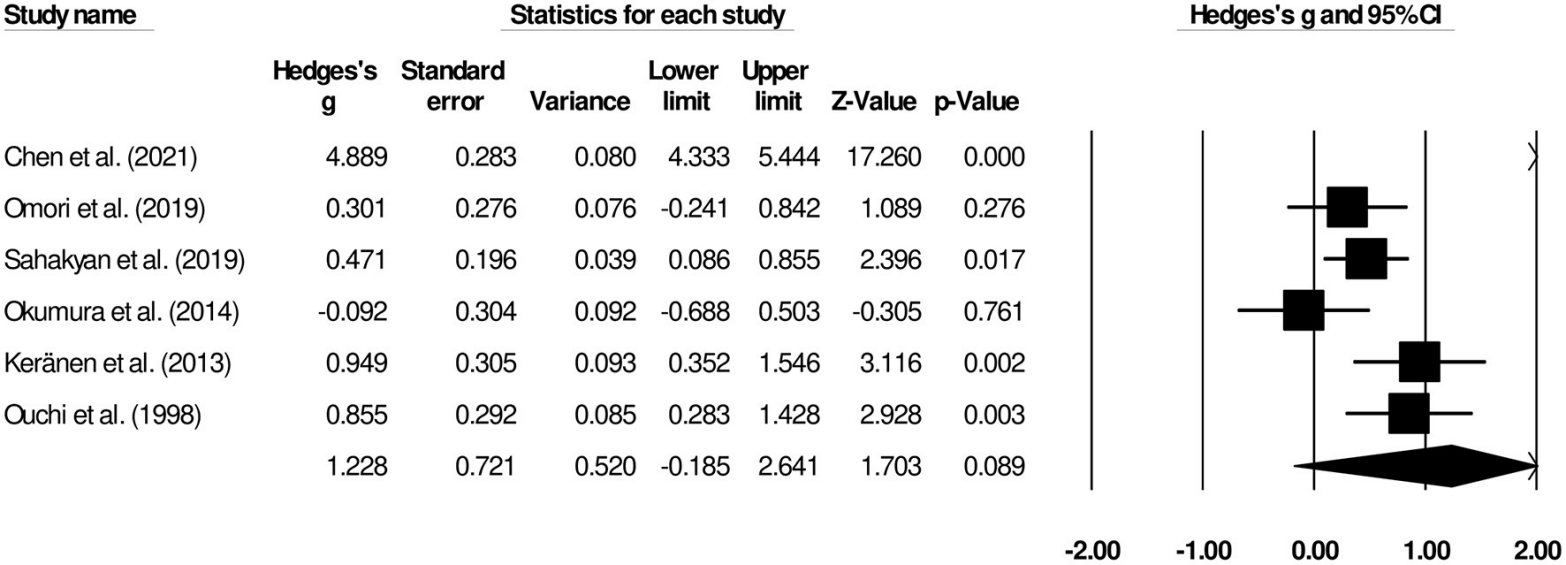
Forest plot for studies comparing the overall survivals for patients undergoing gastrojejunostomy or palliative gastrectomy.

The overall survivals were reported in six studies (9, 14, 16–19). We observed a *large* insignificant effect on overall duration of survival between patients undergoing palliative gastrectomy and gastrojejunostomy (Figure 6) (Hedge's *g*, 1.22; 95% CI, −0.18 to 2.64; *p* = 0.08) with negligible heterogeneity (*I*^2^, 7.09%).

## Discussion

With this systematic review and meta-analysis, we provide comprehensive evidence comparing morbidity- and mortality-related outcomes between patients with metastatic advanced-stage gastric cancer undergoing either palliative gastrectomy or gastrojejunostomy. We observed no statistical significance in terms of the overall survival duration, overall complications, time to oral intake, and overall hospital stay between patients undergoing palliative gastrectomy in those undergoing gastrojejunostomy.

The management of gastric outlet obstruction in patients with metastatic advanced-stage gastric cancer is challenging because of cancer's atypical pathophysiological mechanisms, co-existing morbidities, and manifestations (27–29). Malnutrition, dehydration, and the inability to take palliative medications are common manifestations of patients with gastric outlet obstruction (30), and are associated with poor prognoses for short- and long-term morbidity, mortality, and quality of life (31, 32). Surgical interventions such as gastrojejunostomy and palliative gastrectomy are widely recommended to alleviate the symptoms of patients with gastric outlet obstruction (33). Studies have suggested that these surgical interventions can provide symptomatic relief and improve the complication-free survival of even the patients with dismal prognoses (9). However, whether one of these two interventions results in better postoperative prognostic outcomes than the other for patients with advanced-stage gastric cancer remains unclear (16).

We observed that the included studies had reported different complication rates of these interventions. In a cohort representative of the Japanese population, Omori et al. (14) reported that while 10% of patients who underwent palliative gastrectomy developed complications, none of the patients who underwent gastrojejunostomy exhibited any postoperative complications. The postoperative complications in patients undergoing palliative gastrectomy included abdominal abscess, ileus, leakage, and intraabdominal bleeding. Similarly, Keränen et al. (9) and Ouchi et al. (18) also found higher rates of postoperative complications in patients undergoing palliative gastrectomy. The main complication after palliative gastrectomy in those two studies included re-obstruction of the gastrointestinal pathway and bleeding. On the other hand, Okumura et al. (17), Chen et al. (16), and Sahakyan et al. (19) reported a slightly higher number of postoperative complications in patients undergoing gastrojejunostomy than in those undergoing palliative gastrectomy. In our meta-analysis, we found no difference in terms of risks of complications between patients undergoing palliative gastrectomy and gastrojejunostomy (OR, 1.35). Likewise, in subsequent analyses, we observed that the outcomes of both overall hospital stay (Hedge's *g*, 0.12) and time to oral intake (*g*, 0.62) were insignificantly different between patients undergoing palliative gastrectomy and gastrojejunostomy. Insignificant increase in morbidity that we observed may be due to complications during palliative gastrectomy that are not as frequent with gastrojejunostomy.

We also synthesized the evidence on the overall postoperative survival of patients with advanced-stage gastric cancer. In a retrospective cohort study of 199 patients, Chen et al. (16) reported that the overall survival of patients undergoing palliative gastrectomy (15.9 months; 95% CI, 10.9–20.9 months) was longer than that for gastrojejunostomy (7.7, 5.7–9.6 months). After applying a propensity score matching system for adequately balancing the selection bias, authors further confirmed longer overall survival for patients undergoing palliative gastrectomy (11.8, 7.3–16.3 months) than for those undergoing gastrojejunostomy (8.5, 4.5–12.4 months). Chen et al. (34) recommended the use of palliative gastrectomy due to its high success rate for peritoneal seeding (the most common metastasis pattern in their cohort of patients) management. Similarly, in a cohort of 47 Finnish patients, Keränen et al. (9) found that the palliative gastrectomy improved the survival and the complication-free survival (palliative gastrectomy, 223 days and gastrojejunostomy, 121 days) of patients with advanced gastric cancer to a greater extent than gastrojejunostomy or endoscopic stenting. The authors mentioned that among those three surgical interventions, palliative gastrectomy was the only independent prognostic factor in their multivariate survival analysis. In our meta-analysis, we report no differences in the overall survival outcome of patients with advanced-stage gastric cancer undergoing palliative gastrectomy and gastrojejunostomy (*g*, 1.22).

We are aware of the limitations of our systematic review and meta-analysis. First, we did not pre-register it in a systematic review repository such as PROSPERO York or Joanna Briggs Institute due to the extended registration times during the COVID-19 pandemic. We understand this may raise concerns about the validity of our findings (35), but we decided not to wait more than a year to complete the registration process. Second, the relative paucity of data in the studies included may have created biases in our analysis of the overall times to oral intake and hospital stay lengths. We included data from three different studies in the comparative analysis with small sample sizes (356 for hospital stay length and 305 for time to oral intake). Therefore, we cannot rule out the possibility of a type II error (36). Third, the retrospective nature of each included study is another limitation of the paper. Fourth, we did not conduct extensive subgroup analysis and meta-regression to further elucidate the effects of the various confounders on the final outcomes. Thus, future studies need to address these limitations because risk stratification guidelines for reducing the morbidity- and mortality-related outcomes in patients with metastatic advanced-stage gastric cancer are urgently needed.

In conclusion, our preliminary evidence suggests that there are no significant differences in terms of morbidity and mortality outcomes between patients with metastatic advanced gastric cancer undergoing palliative gastrectomy and outlet obstruction than for those undergoing gastrojejunostomy. Thus, no conclusions can be drawn for the effectiveness of either of the surgical interventions.

## Data Availability Statement

The raw data supporting the conclusions of this article will be made available by the authors, without undue reservation.

## Author Contributions

CL and HF conceived and designed the study and wrote the paper. CL, HF, and WC were involved in literature search and data collection. WC and LC analyzed the data. CL and LC reviewed and edited the manuscript. All authors read and approved the final manuscript.

## Conflict of Interest

The authors declare that the research was conducted in the absence of any commercial or financial relationships that could be construed as a potential conflict of interest.

## Publisher's Note

All claims expressed in this article are solely those of the authors and do not necessarily represent those of their affiliated organizations, or those of the publisher, the editors and the reviewers. Any product that may be evaluated in this article, or claim that may be made by its manufacturer, is not guaranteed or endorsed by the publisher.
