# Supplementary material for: Palliative Gastrectomy vs. Gastrojejunostomy for Advanced Gastric Cancer: A Systematic Review and Meta-Analysis
Source: Front Surg. 2021 Nov 26;8:723065. doi: 10.3389/fsurg.2021.723065 (PMC8661416; doi:10.3389/fsurg.2021.723065)
Supplement: Supplementary file 1 [file Data_Sheet_1.docx]

**Supplementary table 1.** Risk of bias for individual studies based on the Newcastle Ottawa scale

| Study | Selection | | | | Comparability | | Outcome | | | Total |
| --- | --- | --- | --- | --- | --- | --- | --- | --- | --- | --- |
|  | Representative of the exposed cohort | Selection of external cohort | Ascertainment of exposure | Outcome of interest does not present at start | Main factor | Additional factor | Assessment of outcome | Sufficient follow up | Adequacy of follow-up | (9/9) |
| Chen et al. (2021) | + | 0 | + | + | + | 0 | + | + | + | 7 |
| Matsubara et al. (2019) | + | 0 | + | + | + | 0 | + | + | 0 | 6 |
| Omori et al. (2019) | + | 0 | + | + | + | 0 | + | + | + | 7 |
| Sahakyan et al. (2019) | + | 0 | 0 | + | + | 0 | 0 | + | + | 5 |
| Okumura et al. (2014) | + | 0 | + | + | + | 0 | + | + | + | 6 |
| Keränen et al. (2013) | + | 0 | + | + | + | 0 | + | + | + | 7 |
| Ouchi et al. (1998) | + | 0 | 0 | + | + | 0 | + | + | + | 6 |

Supplementary figure 1. Risk of bias according to the Newcastle Ottawa scale for cohort studies
